# The Roles of the Histone Protein Modifier EZH2 in the Uterus and Placenta

**DOI:** 10.3390/epigenomes4030020

**Published:** 2020-09-02

**Authors:** Ana M. Mesa, Cheryl S. Rosenfeld, Geetu Tuteja, Theresa I. Medrano, Paul S. Cooke

**Affiliations:** 1 Department of Physiological Sciences, University of Florida, Gainesville, FL 32610-0144, USA; 2 Grupo de Investigación en Génetica, Mejoramiento y Modelación Animal-GaMMA, Universidad de Antioquia, Medellín 050010, Colombia; 3 Bond Life Sciences Center, Department of Biomedical Sciences, Thompson Center for Autism and Neurobehavioral Disorders, Informatics Institute, and Genetics Area Program, University of Missouri, Columbia, MO 65211, USA; 4 Genetics, Development and Cell Biology, Iowa State University, Ames, IA 50011, USA

**Keywords:** epigenetics, endometrium, stroma, transgenic mice, endometrial cancer, trophoblast, disease

## Abstract

Epigenetic modifications regulate normal physiological, as well as pathological processes in various organs, including the uterus and placenta. Both organs undergo dramatic and rapid restructuring that depends upon precise orchestration of events. Epigenetic changes that alter transcription and translation of gene-sets regulate such responses. Histone modifications alter the chromatin structure, thereby affecting transcription factor access to gene promoter regions. Binding of histones to DNA is regulated by addition or removal of subunit methyl and other groups, which can inhibit or stimulate transcription. Enhancer of zeste homolog 2 (EZH2) is the catalytic subunit of polycomb repressive complex 2 (PRC2) that catalyzes tri-methylation of histone H3 at Lys 27 (H3K27me3) and subsequently suppresses transcription of genes bound by such histones. Uterine EZH2 expression exerts a critical role in development and function of this organ with deletion of this gene resulting in uterine hyperplasia and expression of cancer-associated transcripts. Elucidating the roles of EZH2 in uterus and placenta is essential as EZH2 dysregulation is associated with several uterine and placental pathologies. Herein, we discuss EZH2 functions in uterus and placenta, emphasizing its physiological and pathological importance.

## 1. Introduction

It is becoming increasingly apparent that DNA sequences alone do not dictate gene activity or disease risk. Instead, these processes are regulated by interaction of genes with various factors in the intracellular and extracellular environments. Environmental factors may cause phenotypic effects by inducing mutations in DNA, but commonly they influence gene activity through epigenetic changes. Epigenetics is a term coined by the British developmental biologist Conrad Waddington in 1942, which defines a layer of information that exists beyond that encoded and inherited in the DNA sequence itself [1]. Epigenetic alterations may thus explain how environmental cues alter phenotypic patterns in the absence of genotypic changes [2].

Various epigenetic modifications have been described, including DNA methylation [3] and chromatin remodeling [4], as well as the production of non-coding RNAs (ncRNA) that can induce epigenetic changes. Methylation of the DNA occurs by adding a methyl group (CH_3_) to a cytosine nucleotide preceding a guanine nucleotide (CpG). Areas of the genome that contain high numbers of these sites are known as CpG islands. These CpG islands frequently occur in or near promoter regions of genes [3]. Cytosine methylation generally results in transcriptional repression, as these biomolecular changes may interfere with transcription factors binding and activating their promoter sites. This mechanism promotes silencing by regulating tissue-specific gene expression, X chromosome inactivation, and genomic imprinting [5].

The ncRNA are functional RNAs that are not translated into a protein; some of these have regulatory effects at the transcriptional or post-transcriptional levels. They are classified by size, and there are three major short non-coding RNAs (small interfering RNA (siRNA); microRNA (miRNA), and piwi-interacting RNA (piRNA)), as well as long non-coding RNA (lncRNAs). The miRNAs and siRNAs interact as a regulatory network, often binding to mRNA to promote its degradation, thus inhibiting further translation [6]. siRNAs and lncRNAs can regulate gene expression by recruiting chromatin-modifying proteins that alter chromatin condensation; thus, influencing gene expression [7]. Histone protein modifications are another primary epigenetic mechanism; modification of the histone amino terminal tails affects inter-nucleosomal interactions, thereby altering chromatin arrangement. The degree of compaction of the chromatin modulates the accessibility of the DNA to the transcriptional machinery; therefore, compacted chromatin inhibits gene expression [8].

Within chromosomes, DNA is encased by members of a protein family called histones to form nucleosomes. Chromatin functionality is modified by assembly and disassembly of these nucleosomes, and histones are key in this [9]. Histones are susceptible to various post-translational modifications (PTMs), such as methylation, acetylation, phosphorylation, and ubiquitination, on their N- and C-terminal tails. The nature and amino acid location of such modifications modulate the degree of tightness or laxity of the chromatin, in turn affecting gene expression [10]. We will focus on histone protein methylation as one way EZH2, the epigenetic modifier being considered, acts to induce such modification.

Methylation commonly occurs on histones H3 and H4 on specific lysine (K) and arginine (A) residues. Lysine (K) can be mono, di and tri-methylated, which dictates the biological outcome [11]. In contrast to typically suppressive effects of DNA methylation, the net result of histone methylation on gene expression is variable depending on the specific histone protein and amino acid residue modified. For instance, lysine methylation on H3K9 (histone 3, lysine 9), H3K27, or H4K20 can inhibit transcription. Conversely, methylation on H3K4 and H3K36 stimulates transcription [12]. Histone modifications also interact with each other and with DNA methylation [13].

Enzymes that catalyze these fundamental epigenetic alterations are identified as “writers” and “erasers” [14]. Writers are enzymes that add PTMs to histones and are classified according to their actions, such as histone methyltransferases (HKMTs) and histone acetylases (HATs). Erasers remove specific PTMs, including histone demethylases (HDMTs) and histone deacetylases (HDACs) [15].

**Figure 1.**
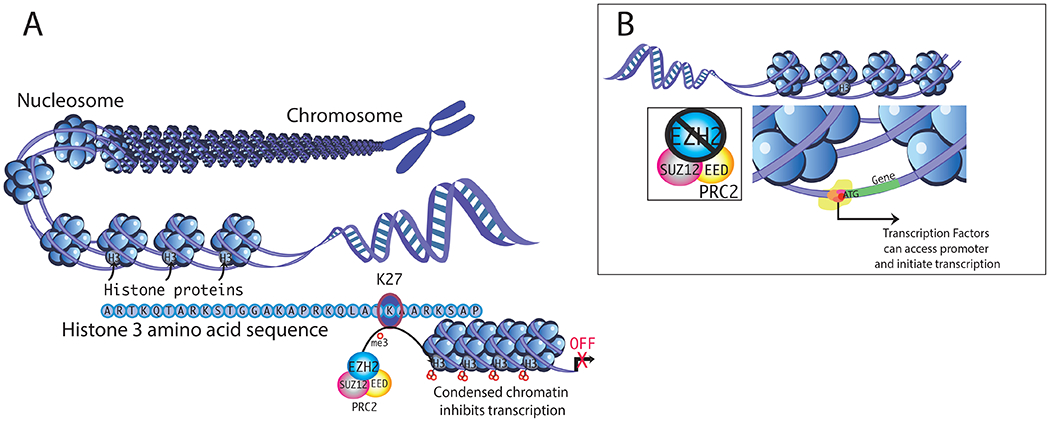
(**A**). Mechanism of transcriptional silencing by histone 3 methylation. Methylation at histone H3 lysine-27 (H3K27) is one factor that can induce chromatin condensation, repressing gene transcription by limiting access of the transcription machinery to gene promoters. Polycomb repressive complex 2 (PRC2) contains multiple subunits. Enhancer of zeste homolog 2 (EZH2) is the enzyme that promotes methylation of H3K27. (**B**). In the absence of EZH2, chromatin is configured more accessibly, which promotes transcription.

Polycomb repressive complex 1 and 2 (PRC1 and PRC2) function as epigenetic repressors [16]; PRC2 is a histone methyltransferase complex that tri-methylates histone 3 on lysine 27 to produce H3K27me3 [17]. H3K27me3 is a hallmark of transcriptional repression [11]. Enhancer of zeste homolog 2 (EZH2) is the catalytic subunit of PRC2 that governs trimethylation of H3K27 (Figure 1) [18].

Dysregulation of H3K27 methylation is associated with multiple pathologies, including tumorigenesis and metastasis [19]. Neoplastic changes where EZH2 overexpression has been implicated include endometrial [20,21], colorectal [22], breast [23], and pancreatic cancer [24]. Changes in uterine EZH2 expression in women are also associated with endometrial hyperplasia, endometriosis, and uterine fibroids [20,21], suggesting that this enzyme is required for epigenetic programming in uterine epithelium and stroma.

In this review, we will discuss potential roles of EZH2 in regulating normal physiological responses in the uterus and the placenta, which forms an intimate association with the uterus during pregnancy. To help understand EZH2’s role in the uterus and other organs, transgenic mouse models have been created in the past few years, and the resulting phenotypic changes in these mice will be described. Lastly, we will consider the role of EZH2 in uterine diseases.

## 2. Actions of EZH2

Three primary mechanisms of EZH2 action have been characterized. In the canonical pathway, the action of the PRC2 complex requires EZH2, which results in trimethylation of H3K27, i.e., H3K27me3 formation. The closely related PRC1 family ubiquitinates histones and consists of heterogeneous variants, which contain CBX (chromobox-containing protein) and signal through the canonical pathway or lack CBX proteins and signal through non-canonical pathways. PRC1 plays important roles during embryology but also disease formation. There is also recent evidence that PRC1 can function as a transcriptional activator [25]. For further information on PRC1, Khan et al. provide a comprehensive review describing the interplay of these two polycomb complexes [26].

The core components of PRC2 are EZH2, embryonic ectoderm development (EED), suppressor of zeste 12 (SUZ12), and retinoblastoma (Rb)-associated protein 46/48 (RbAp46/48) [16]. EZH2 is the catalytic subunit, and it associates with EED to operate as a scaffold protein (Figure 1) [27]. *Ezh1* is believed to have arisen by gene duplication from *Ezh2. Ezh1*, in some cases, may compensate for the loss of *Ezh2*, and can also potentially have a role in cancer. However, our focus is on EZH2, as there is strong evidence to suggest that it can be affected by xenoestrogens and dysregulation of EZH2 leads to a variety of cancers, including uterine cancer, as detailed below [28]. Wassef et al. [29] provide further information on EZH1.

The SUZ12 protein aids nucleosome recognition [30] and RbAp46/48 contributes to histone binding [31]. The PRC2 complex also includes proteins such as AE Binding Protein 2 (AEBP2), polycomb-like (PCLs) proteins, and jumonji, AT rich interactive domain 2 (JARID2) [31]. Interaction of PRC2 with DNA often requires key transcription factors. For example, the transcription factor Yin Yang 1 (YY1) directly interacts with EZH2 and recruits it to specific genome sites to regulate gene expression. Both EZH2 and YY1 are associated with repressing tumor suppressor APC in endometrioid endometrial carcinomas [32].

Phosphorylation of EZH2 on serine 21 by protein kinase B (AKT) inhibits PRC2-mediated H3K27 enzymatic activity. However, this phosphorylation is seemingly necessary for PRC2-independent functions of EZH2 [33]. Threonine residues 345 and 487 on EZH2 are targets of phosphorylation by cyclin-dependent kinase 1 (CDK1) to facilitate binding to ncRNA, promoting PRC2 recruitment [34]. For the degradation of EZH2, JAK2 phosphorylates EZH2 at tyrosine 641 [32]. EZH2 can influence gene expression by methylation of non-histone factors, such as the signal transducer and activator of transcription 3 (STAT3), GATA Binding Protein 4 (GATA4), talin1, and RAR-related orphan receptor alpha (RORα). These non-histone methylations still are carried out by EZH2 through the PRC2 complex [35].

Lastly, EZH2 can alter gene expression in a PRC2-independent manner, which entails the methylation of non-histone targets or interaction with other transcription factors to activate downstream genes. For example, EZH2 functions as a co-activator of estrogen receptor 1 (ESR1; also known as ERa) and promotes the transcription of its target genes. Thus, EZH2 inhibits gene expression by altering chromatin structure, but it can also act as a coactivator and promote transcription. Whether EZH2 acts to repress or stimulate transcription largely relates to its association with other proteins. EZH2 regulation itself occurs through post-translational modifications, including the phosphorylation of various amino acids within the protein.

## 3. Transgenic Models to Study Global and Uterine Roles of EZH2

The global deletion of *Ezh2* induces early embryonic lethality. The null mouse embryos implant in the uterus but fail to correctly develop between gestation day (GD) 6.5 and 8.5. Normal embryonic germ layers fail to differentiate, and abnormal development of mesoderm cells in extraembryonic cells are observed [36]. Determining EZH2’s role in specific organs or tissues, therefore, requires generation of conditional knockouts where EZH2 is deleted specifically in those organs or cell types.

**Figure 2.**
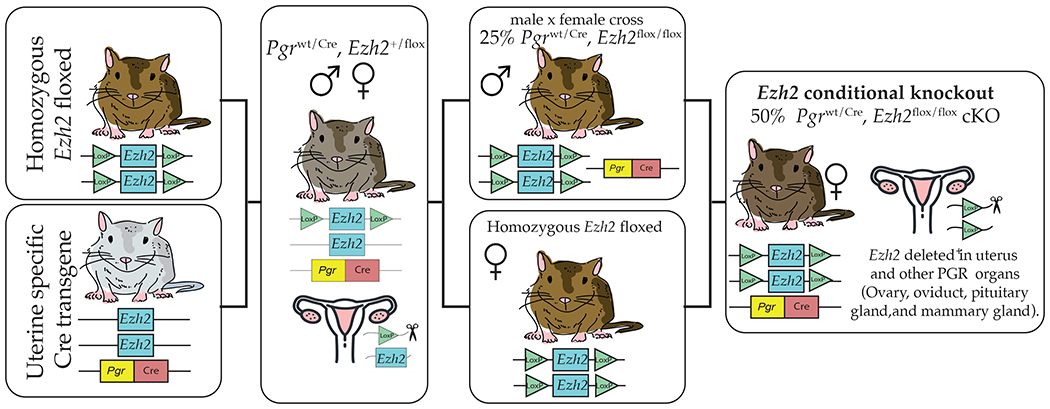
To generate *Ezh2*cKO, mice in which both copies of the *Ezh2* gene were floxed (*Ezh2*^flox/flox^) were crossed with mice expressing cre recombinase driven by the progesterone receptor promoter *Pgr*^wt/Cre^. The first cross incorporates the cre recombinase that generates an EZH2 deletion in one of the two alleles. Offspring of this cross were mated to obtain males expressing (*Pgr*^wt/Cre^, *Ezh2*^flox/flox^). These males were then mated with *Pgr*^wt/wt^, *Ezh2*^flox/flox^ females. Half of the females produced by this cross are a *Pgr*^wt/Cre^, *Ezh2*^flox/flox^ expressing cre recombinase and with both EZH2 alleles floxed (*Ezh2*cKO). The other half are *Pgr*^wt/wt^, *Ezh2*^*flox/flox*^ WT controls.

To determine the role of EZH2 in the uterus, we have generated a conditional knockout mouse lacking EZH2 in progesterone receptor (PGR)-expressing cells in the uterus and other organs, such as the ovary, oviduct, pituitary gland, and mammary gland. [37]. To produce this transgenic line, mice with two loxP sites flanking exons 14–15 within the *Ezh2* gene [38], were obtained commercially. Transgenic mice expressing Cre recombinase under the control of the progesterone receptor (*Pgr*) promoter [37] provide a powerful tool to obtain Cre expression in most but not all uterine cell types. Female mice with a conditional deletion of the *Ezh2* gene were generated as described in Figure 2. To obtain insights into uterine roles of EZH2, RNA-seq analysis was performed using *Ezh2*cKO and WT female mice receiving vehicle (V) or 17β–estradiol (E2) [39]. Conditional loss of EZH2 in the uterus upregulated genes associated with uterine diseases, including keratin 5 (*Krt5*), keratin 15 (*Krt15*), the maternally-imprinted gene- *H19*, leucine-rich repeat-containing G-protein-coupled receptor 5 (*Lgr5*), and cell division cycle associated 5 (*Cdca5*). Fang et al. [40] also showed that KRT5, KRT6A, and KRT14 protein and mRNA expression were elevated in *Ezh2*cKO uteri relative to WT controls [40]. Based on these expression patterns and enhanced uterine epithelial proliferation in these mice, it was postulated that EZH2 suppresses differentiation of basal-like cells and consequently restricts uncontrolled uterine epithelial proliferation. Weighted gene co-expression network analysis (WGCNA) to link gene expression changes to uterine epithelial proliferation confirmed that estrogen, PI3K/AKT, and other signaling pathways, as well as histone protein modifications, are likely altered in *Ezh2*cKO mice, and may be involved in the uterine epithelial proliferation seen even following ovariectomy in these animals.

## 4. Roles of EZH2 in the Normal Uterus and Uterine Pathologies

Disrupted EZH2 expression has been implicated in several uterine diseases, including endometriosis, uterine fibroids, endometrial cancer, and other proliferative disorders [20,21,41–45]. Collectively, these findings suggest that this epigenetic modifier regulates various hyperproliferative diseases involving both endometrial epithelium and stroma. Scant information, though, is available on specific EZH2 roles in regulating normal non-gravid and gravid uterine physiology, as well as how it might change throughout the estrous or menstrual cycles. Epithelial EZH2 expression is upregulated by E2 and inhibited by progesterone in adult uteri from wild-type ovariectomized mice [46], but it is unclear if such hormones may also regulate post-translational modifications, namely phosphorylation, that alter EZH2 activity. Our main observation in *Ezh2*cKO uteri supports a fundamental role for this protein in restraining uterine epithelial proliferation, as these mice had uterine luminal and glandular epithelial hyperplasia [46]. Similar results were reported in the *Ezh2*cKO model by Fang et al. [40], who additionally reported increased expression of tumor markers and uterine epithelial stratification in their transgenic mouse line.

The EZH2 mutations found in endometrial and other cancers are predominantly gain of function mutations [47–49]. Amounts of EZH2 frequently correlate with tumor growth, development, and prognosis [50]. In the endometrium and other organs, EZH2 is a key player in epithelial-mesenchymal transition (EMT) [51]. This process consists of biochemical transformations that convert polarized epithelial cells into cells that morphologically and functionally resemble mesenchymal cells in terms morphology and other cellular properties. Mesenchymal cells exhibit enhanced migratory capacity, and such cells are resistant to apoptosis [52]. This EMT process is observed during embryonic development, wound healing, and cancer metastasis [53]. Zhang et al. [51] describe an upregulation of EZH2 and other PRC2 proteins in endometrium from women with endometriosis; knocking down or inhibiting EZH2 reduced cell migratory and invasive cell markers. Colon-Caraballo et al. [20] have shown high nuclear EZH2 immunostaining and increased H3K27me3 levels in endometriotic lesions compared to normal endometrium. Furthermore, pharmacological inhibition of EZH2 with GSK343 reduced global H3K27me3 levels, specifically in diseased endometriotic cells, and this treatment inhibited proliferation and migration of these endometriotic cells [54]. Gu et al. [55] quantitated EZH2 expression in endometrial carcinoma patients and found EZH2 expression was correlated with tumor invasiveness. In contrast, uterine leiomyomas induced by genistein have decreased EZH2 andH3K27me3 marks [41].

**Figure 3.**
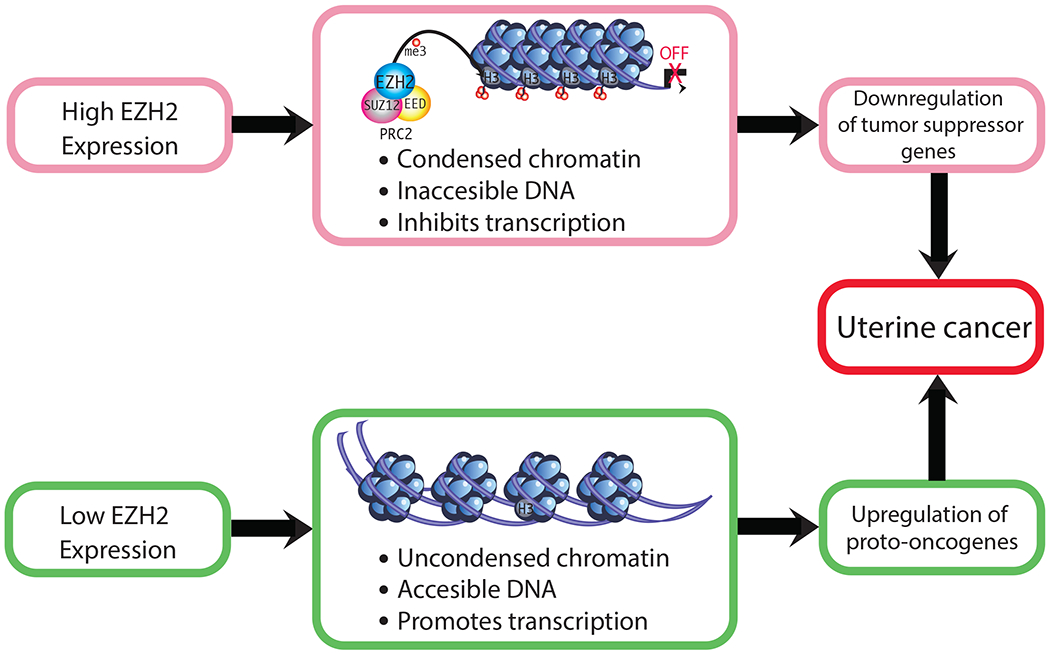
Mechanisms by which altered EZH2 expression could increase cancer susceptibility. Changes in the expression of EZH2 can downregulate the expression of tumor suppressor genes [57] and upregulate oncogenes [58,59].

These studies indicate that EZH2 dysregulation increases risk for cancer or other proliferative diseases, depending upon which histone protein marks and corresponding genes are affected by this epigenetic modifier. For instance, if EZH2-induced methylation of H3K27 normally represses a transcription factor or proto-oncogene, EZH2 loss may result in expression of that gene and downstream genes. For proto-oncogenes, loss of EZH2 may lead to unrestrained transcription of these genes, with resulting neoplastic transformation. However, specific ones related to endometrial cancer remain to be identified. Conversely, for tumor suppressor genes, EZH2 overexpression could suppress these genes and increase susceptibility to tumorigenic changes. For example, EZH2 overexpression in endometrial cancer causes a downregulation of the tumor suppressor gene *ARID1A*. The reduction in this tumor suppressor gene presumably leads to unrestrained expression of cancer-promoting genes [56]. Thus, both the under- and over-expression of EZH2 can be associated with increased cancer risk (Figure 3). A critical question is whether altered EZH2 expression is in and of itself sufficient to induce uterine cancer or whether upregulation of this gene is merely associated with endometrial cancer. Its potential role in this disease remains to be definitively established.

While we have not examined these mice at later ages to determine if their uterine pathologies become more severe, it is clear that women with endometrial hyperplasia are at risk for endometrial cancer [40,46]. Transgenic animal models, epidemiological studies, and cell culture approaches will provide mechanistic insight into EZH2’s role in the normal and pathological uterus. While other specific targets of EZH2 in uterine tissue remain to be determined, gene targets of EZH2 in breast [60], and prostate cancer cell types [61] have been reported. Noteworthy identified targets of EZH2 are the promoters for androgen receptor (AR) [61], E-cadherin and Forkhead box transcription factor-1 (*FOXO1*) [62]. Colón-Caraballo et al. [54] identified H3K27me3 enrichment in the promoter regions of *ESR1, CDH1*, and *PGR*, using human endometriotic tissues.

## 5. Role of EZH2 in the Placenta

The placenta is a transient organ that is essential during pregnancy, in that it allows for communication between mother and fetus and exchange of nutrients, gases, and waste. Defects in placental development lead to a number of disorders, such as preeclampsia, recurrent miscarriage, stillbirth, and intrauterine growth restriction, that impact the mother, embryonic or fetal growth, and/or later offspring health [63]. The placenta is composed of heterogeneous cell types that are important for anchoring the fetus to the uterine wall, hormone secretion, nutrient transfer, immune regulation, and other functions. Placental cell types are reviewed in detail elsewhere [64,65]. In order to study placental function in vivo, mouse and rat models are frequently utilized. Like humans, rodents have a hemochorial placenta [66], with maternal blood in direct contact with fetal placental cells. Valuable insights into human placental development have been gained by genetic manipulation of rodents [67].

A conditional knockout mouse model has been created to help understand the role of EZH2 in the mouse placenta. Nugent et al. [68] found that O-linked *N*-acetylglucosamine transferase (*Ogt*), an X-linked gene that has low expression in placentas of male fetuses under maternal stress conditions [69], contributes to sex-specific H3K27me3 profiles in trophoblast cells, and is associated with neurodevelopmental vulnerability in males [68]. The investigators, therefore, became interested in determining if reduced placental H2K27me3 levels, resulting from placental *Ezh2* deletion, combined with prenatal stressors (e.g., overnight noise or light, restraint stress, odor exposure, or cage changes), would result in similarly impaired neurodevelopment in females. To generate placenta-specific *Ezh2* conditional knockout mice, a previously developed transgenic mouse model was used [70] that is similar to the one we and other used to make an *Ezh2* conditional knockout in the uterus. this model, a regulatory sequence that is responsible for placenta-specific gene expression of aromatase P450 (CYP19), already defined previously in the human, was used to drive Cre expression [70]. Using this transgenic model, *Ezh2* expression was ablated from the trophoblast lineages of the placenta by GD 11.5, the age by which *CYP19*-Cre is expressed in all trophoblast cells. While the trophoblast-specific *Ezh2*cKO did not impact bodyweight or cause neuroendocrine dysregulation in females, the combination of trophoblast-specific *Ezh2* knockout and prenatal stress led to later pathological changes [68]. Specifically, female knockout mice had increased body weight at weaning and through adulthood compared to wild-type mice, and had increased corticosterone responses following restraint stress. In summary, placental *Ezh2* deletion made females more vulnerable to prenatal stress.

As noted above, the *CYP19*-Cre transgene is expressed in all cells derived from trophoblast stem cells by GD 11.5. Therefore, it is unclear what the impact would be if *Ezh2* expression were reduced in trophectoderm cells of the blastocyst that give rise to the chorionic portion of the placenta. As the placenta is composed of multiple trophoblast types with diverse functions, the impact of deleting *Ezh2* in specific trophoblast cell populations, such as invasive trophoblast cells that are important for anchoring the placenta to the uterine wall, spongiotrophoblast and trophoblast giant cells that are important for steroid and peptide hormone production, and glycogen trophoblasts that serve as an energy reserve for the placenta [66], would also be interesting. Furthermore, the study did not determine how conditional placental *Ezh2* deletion affected the histology of this organ and trophoblast differentiation. Placenta-specific *Ezh2c*KO mice may have defects in placenta structure, weight, or nutrient transport functions that could impact females in ways not assessed in this study. Similarly, different types of maternal stressors could lead to different phenotypes observed in the placenta and resulting offspring.

Excess glucocorticoids in pregnancy is another stressor that can lead to neuroendocrine dysregulation. In the placenta, 11β-hydroxysteroid dehydrogenase 2 (11β-HSD2) inactivates glucocorticoids, contributing to establishment of the glucocorticoid barrier, thus regulating fetal glucocorticoid exposure [71–73]. The placental glucocorticoid barrier is composed of syncytiotrophoblast cells, which generally form the interface between the mother and fetus, allowing the gas and nutrient exchange necessary for normal fetal growth [65]. Therefore, while syncytiotrophoblast have high 11β-HSD2 expression, their cytotrophoblast precursors do not. Zuo et al. [74] were interested in understanding the mechanisms associated with 11β-HSD2 repression prior to cytotrophoblast fusion to form the syncytiotrophoblast (syncytialization) [74]. This group found that in both early and term villous tissue, EZH2 was strongly expressed and localized to cytotrophoblast nuclei, but only weakly expressed in syncytiotrophoblasts. This expression pattern was opposite to that observed for 11β-HSD2 [74]. Furthermore, during syncytialization, EZH2 expression and H3K27me3 expression both decreased. Additional experiments demonstrated that upon syncytialization, 11β-HSD2 expression is induced following inactivation of the pRB-E2F1-EZH2 pathway caused by hCG-cAMP-PKA pathway mobilization [74]. EZH2 inactivation during syncytialization likely contributes to activation of a number of genes important for placental metabolic function besides 11β-HSD2. Similarly, EZH2 activation could repress cytotrophoblast genes, and low cytotrophoblast EZH2 expression could alter placental development and result in placenta-associated pregnancy disorders. Direct targets of EZH2 have been identified in human trophoblast cell lines; these include *RND3* [75], and *HSD11B2* [74]. The interaction of EZH2 with *ANGPTL4* [76] was reported in human cytotrophoblasts.

Lv et al. [77] found that EZH2 expression is lower in first trimester villous tissues from patients with recurrent miscarriage compared to control patients [77]. Abnormal trophoblast invasion is associated with recurrent miscarriage [78], leading the authors to investigate the role of EZH2 in trophoblast invasion using the JAR choriocarcinoma cell line. In addition to determining that EZH2 promotes trophoblast invasion in JAR cells, the authors also found that EZH2 represses a tumor repressor gene that inhibits EMT, caudal type homeobox 1 (CDX1) [77]. A role for EZH2 in promoting trophoblast EMT is consistent with its known role in promoting this process in endometriosis [51]. Overall, this study suggested that EZH2 could be a therapeutic target for treating recurrent miscarriage. However, most of the mechanistic studies were carried out in a choriocarcinoma cell line, due to the difficulty in obtaining primary cells. While such cells may retain properties similar to trophoblast cells, they are a cancer cell line with increased proliferation and other properties different from non-cancerous counterparts. Given the recent description of how to maintain and propagate human trophoblast stem cells in culture and differentiate such cells into extravillous cytotrophoblast and syncytiotrophoblast [79], it would be of interest to repeat these experiments with this more physiological culture system.

## 6. Conclusions

EZH2 is a critical epigenetic modifier acting predominately through the PRC2 complex to affect the H3K27me3 histone protein methylation mark. Uterine EZH2 plays a critical role in regulating epithelial proliferation; both under and overexpression of this protein may result in hyper-proliferative conditions. Similarly, EZH2 dysregulation is linked to placenta-associated pregnancy disorders. The *Ezh2*cKO mice provide an experimental model to elucidate the complex roles of EZH2 in uterus and placenta. Although select genes affected by EZH2 have been identified in these organs, the global gene targets remain uncertain. Therefore, these transgenic mouse models, coupled with advanced ’omics technology, such as spatial transcriptomics and chromatin immunoprecipitation sequencing, will further our understanding of how loss of *Ezh2* in the uterus and placenta affects transcriptomic profiles in specific cell-types. Such knowledge will have important clinical ramifications in understanding uterine and placental regulation by EZH2 in health and disease.

## Abbreviations

AEBP2: AE Binding Protein 2
AKT: protein kinase B
CBX: chromobox-containing protein
CYP19: aromatase P450
11ß-HSD2: 11ß-hydroxysteroid dehydrogenase 2
*Cdca5*: cell division cycle associated 5
CDX1: caudal type homeobox 1
E2: 17β-estradiol
EED: embryonic ectoderm development
EMT: epithelial mesenchymal transition
EREs: estrogen response elements
ESR1: estrogen receptor 1
EZH2: enhancer of zeste homolog 2
*Ezh2*cKO: Ezh2 conditional knockout
GATA4: GATA Binding Protein 4
GD: gestation day
HDACs: histone deacetylases
HDMTs: histone demethylases
HATs: histone acetylases
JARID: jumonji, AT rich interactive domain 2
*Krt5/Krt15/Krt6A/Krt14*: keratin 5, 15, 6A and 14
*Lgr5*: leucine-rich repeat-containing G-protein-coupled receptor 5
lncRNAs: long non-coding RNA
miRNA: microRNA
ncRNA: non-coding RNAs
PGR: progesterone receptor
piRNA: piwi-interacting RNA
PCL: polycomb-like
PND: postnatal day
PRC1 and PRC2: polycomb repressive complex 1 and 2
PTM: post-translational modifications
RbAp46/48: retinoblastoma (Rb)-associated protein 46/48
RORα: RAR-related orphan receptor alpha
siRNA: small interfering RNA
STAT3: signal transducer and activator of transcription 3
SUZ12: suppressor of zeste 12
TNM: tumor necrosis and metastasis (method to classify tumors)
V: vehicle
WGCNA: weighted gene co-expression network analysis
YY1: Yin Yang 1
